# Advancements in technology for characterizing the tumor immune microenvironment

**DOI:** 10.7150/ijbs.92525

**Published:** 2024-03-25

**Authors:** Honglin Yan, Xianli Ju, Aoling Huang, Jingping Yuan

**Affiliations:** Department of Pathology, Renmin Hospital of Wuhan University, Wuhan, Hubei 430060, P.R. China.

**Keywords:** Tumor immune microenvironment, Cytometry-based techniques, Multiplexing imaging techniques, RNA sequencing, Artificial intelligence

## Abstract

Immunotherapy plays a key role in cancer treatment, however, responses are limited to a small number of patients. The biological basis for the success of immunotherapy is the complex interaction between tumor cells and tumor immune microenvironment (TIME). Historically, research on tumor immune constitution was limited to the analysis of one or two markers, more novel technologies are needed to interpret the complex interactions between tumor cells and TIME. In recent years, major advances have already been made in depicting TIME at a considerably elevated degree of throughput, dimensionality and resolution, allowing dozens of markers to be labeled simultaneously, and analyzing the heterogeneity of tumour-immune infiltrates in detail at the single cell level, depicting the spatial landscape of the entire microenvironment, as well as applying artificial intelligence (AI) to interpret a large amount of complex data from TIME. In this review, we summarized emerging technologies that have made contributions to the field of TIME, and provided prospects for future research.

## Introduction

The tumor microenvironment (TME) is a complex ecosystem that comprises tumor cells, stromal cells, immune cells, and cytokines. In recent years, research has begun to elucidate the role of the tumor immune microenvironment (TIME) in the development and progression of cancer. The TIME includes tumor-antagonizing immune cells, such as effector T cells, natural killer (NK) cells, and M1-polarized macrophages; tumor-promoting immune cells, such as regulatory T cells (Tregs) and myeloid-derived suppressor cells (MDSCs); and signaling molecules or factors, such as checkpoint receptors and their corresponding ligands 1, 2.

Conventional technologies, such as flow cytometry and immunohistochemistry (IHC), have been instrumental in elucidating the composition and molecular characteristics of the TIME as well as developing clinical methodologies to better treat tumors. Furthermore, significant resources have been devoted to analyzing the spatial structure of the TIME. The spatial distribution of the TIME can be summarized by four aspects: location—namely the spatial distribution and cell proportion of various immune cells in the TIME; distance between immune cells and their nearest neighbors; spatial distribution of cell-cell interactions at the level of antigen recognition, such as immune regulators; and special spatial patterns, such as the spatial characteristics of activation or inhibition of immune cells with molecular and morphological characteristics 3, 4.

In the past few years, major advances have already been made in characterizing the TIME at a considerably elevated degree of throughput, dimensionality, and resolution. Notably, recent advances in techniques, such as imaging mass cytometry (IMC), multiplex immunohistochemistry/immunofluorescence (mIHC/IF), single-cell RNA sequencing (scRNA-seq), and spatial RNA sequencing (spRNA-seq) have been employed to delineate the landscape of the TIME and unravel the heterogeneity of its composition, function, and immune cell distribution within the TIME 5-8. In this review, we highlighted the advancements of cytometry-based techniques (including flow cytometry, mass cytometry, and IMC), multiplexing imaging techniques (such as mIHC/IF), RNA sequencing (from bulk, single-cell to spatial RNA sequencing), and artificial intelligence (AI) in characterizing the TIME.

## Overview of the TIME

**Figure 1:**
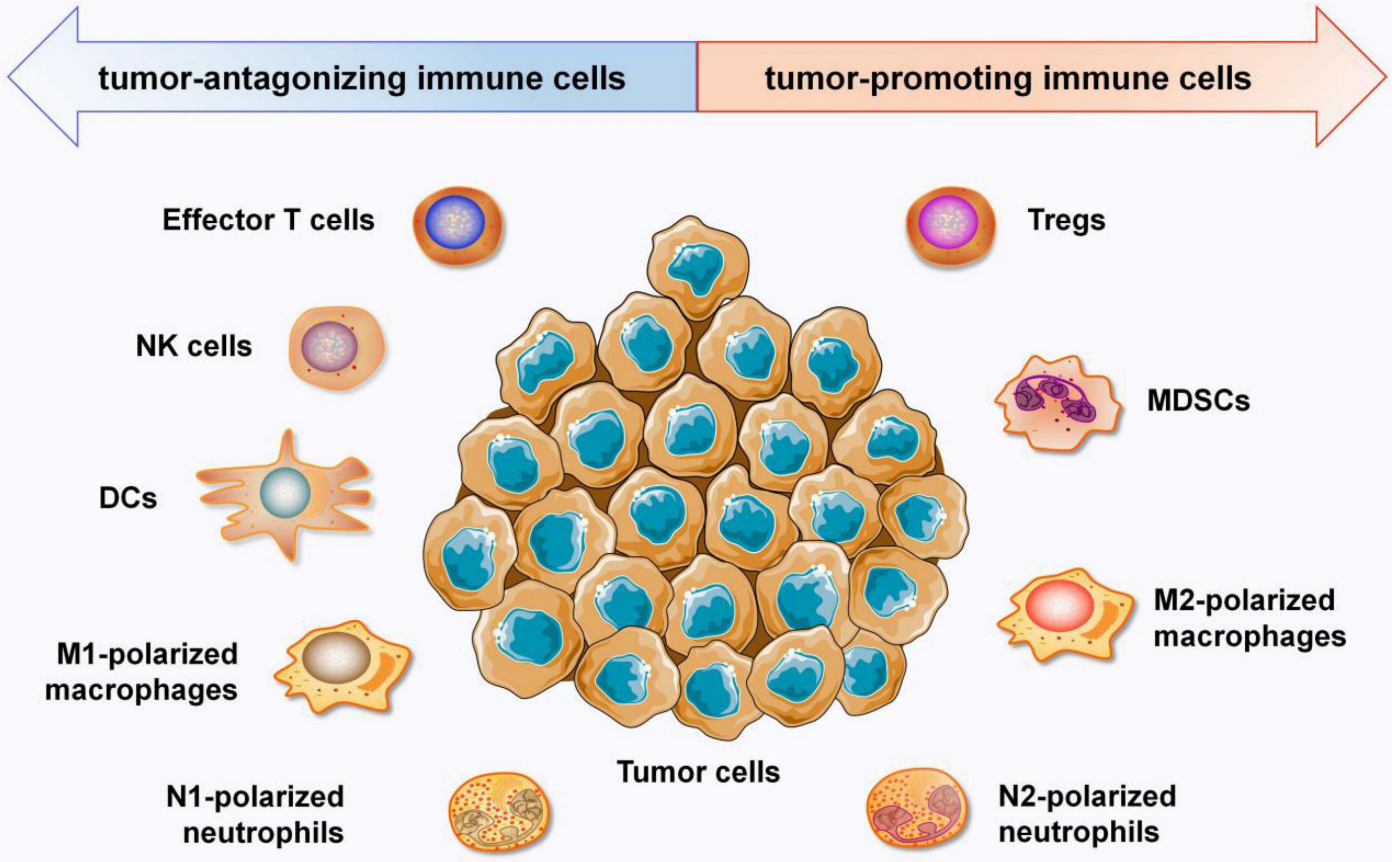
** Overview of the the immune components that constitute the TIME.** Immune cells in the TIME are divided into two categories: tumor-antagonizing immune cells and tumor-promoting immune cells. Effector T cells, NK cells, DCs, M1-polarized macrophages and N1-polarized neutrophils function as the tumor-antagonizing immune cells, while Tregs, MDSCs, M2-polarized macrophages and N2-polarized neutrophils function as tumor-promoting immune cells.

In recent years, the TIME has attracted increasing interest due to its crucial roles in immunotherapy. The TIME consists mostly of tumor cells; extracellular matrix; cytokines; and distinct immune cell populations, such as T lymphocytes, tumor associated macrophages (TAMs), neutrophils, MDSCs, NK cells, dendric cells (DCs) and other immune cells 5. Immune cells in the TIME are roughly divided into two categories: tumor-antagonizing immune cells and tumor-promoting immune cells (Figure 1). Effector T cells, NK cells, DCs, M1-polarized macrophages and N1-polarized neutrophils function as tumor-antagonizing immune cells, while Tregs, MDSCs, M2-polarized macrophages, and N2-polarized neutrophils function as tumor-promoting immune cells. It is important to note that the role of immune cells within the TIME can vary depending on the specific disease, tumor type, and individual patient factors.

T lymphocytes, also known as T cells, are a critical component of the adaptive immune system 9. They play a vital role in recognizing and eliminating infected cells, foreign invaders, and cancerous cells. CD4^+^ helper cells (Th cells) and CD8^+^ cytotoxic T lymphocytes (CTLs) are the main subsets of T lymphocytes 9, 10. Treg cells are a specialized subset of T lymphocytes that act as immune "brakes" by suppressing the activity of other immune cells, such as effector T cells, B cells, and antigen-presenting cells 11, 12. The suppressive activity of Tregs within the tumor microenvironment can hinder anti-tumor immune responses and promote immune evasion by cancer cells 11, 12. DCs and NK cells are tumor-antagonizing immune cells. DCs bridge the innate and adaptive immune responses by capturing and presenting antigens to T cells, playing a critical role in initiating and shaping adaptive immune responses 13, 14, while NK cells are innate immune cells that eliminate infected and cancerous cells by releasing cytotoxic molecules and cytokines 15. In the context of cancer, certain immune cells have the ability to assume dual roles. For example, macrophages are vital components of the innate immune system and can be dynamically polarized to produce distinct functional phenotypes by various signals within the microenvironment, namely M1-polarized (classically activated) macrophages and M2-polarized (alternatively activated) macrophages, which are suggested to have opposite roles in tumor development 16-18. M1-polarized macrophages display a tumoricidal phenotype and are involved in promoting anti-tumor immune responses. They release pro-inflammatory cytokines and generate reactive oxygen species (ROS) and reactive nitrogen species (RNS) to eliminate tumor cells. In contrast, M2-polarized macrophages exhibit a pro-tumorigenic phenotype and contribute to tumor progression. They secrete anti-inflammatory cytokines and growth factors, which subsequently promote tumor cell growth, angiogenesis, and tissue remodeling. Similar to the tumoricidal and pro-tumorigenic macrophages, tumor-associated neutrophils (TANs) are also divided into anti-tumorigenic N1 and pro-tumorigenic N2 phenotypes 19-21. N1-polarized TANs exhibit anti-tumorigenic properties by stimulating the production of ROS, tumor necrosis factor (TNF), and intercellular adhesion molecule (ICAM)-1. Additionally, N1-polarized TANs can activate various innate and adaptive immune cells, further enhancing their anti-tumor functions. These activities collectively contribute to the suppression of tumor growth and metastasis. On the contrary, N2-polarized TANs display a pro-tumorigenic phenotype and are characterized by the secretion of pro-angiogenic factors and enzymes involved in extracellular matrix (ECM) remodeling. Consequently, N2-polarized TANs promote angiogenesis and metastasis, which directly or indirectly support tumor growth and the dissemination of tumor cells. Among tumor-promoting immune cells, MDSCs exert their immune-suppressive function through multiple mechanisms 22-24. They inhibit T cell responses by depleting essential nutrients, producing inhibitory cytokines like interleukin-10 (IL-10) and transforming growth factor-beta (TGF-β), as well as inducing Tregs. MDSCs can also directly suppress the activity of NK cells and DCs. Moreover, MDSCs promote tumor growth and metastasis by creating an immunosuppressive microenvironment, supporting angiogenesis, and facilitating tumor cell invasion and extravasation.

The infiltration of immune cells into the tumor microenvironment leads to significant alterations in the immune landscape of the tumor. Understanding the unique characteristics and subclasses of TIME present in patients' tumors will improve the ability to predict patient treatment and guide prognosis 2. Recently, significant progress has been made in analyzing the functional status and spatial distribution of immune cells to define subtypes of the TIME. Wang et al. 25 characterized the TIME into three distinct states: active TIME (A-TIME), equilibrated TIME (E-TIME), and suppressive TIME (S-TIME). A-TIME is characterized by a robust infiltration of CD8^+^ effector T cells and M1-polarized macrophages. Patients with A-TIME generally exhibit more favorable clinical outcomes. On the contrary, an S-TIME is associated with an increased presence of Th cells, Tregs, and M2-polarized macrophages, while the E-TIME state represents a balance between immune effector cells and immunosuppressive cells. Similarly, Binnewies et al. 2 described three broad classes of TIME: infiltrated-excluded (I-E) TIME, infiltrated-inflamed (I-I) TIME, and tertiary lymphoid structures (TLS) TIME. The I-E TIME features a wide distribution of immune cells but lacks CTLs in the tumor core and has been hypothesized to be poorly immunogenic or “cold” 2, 26. Meanwhile, the I-I TIME is characterized by high infiltration of CTLs expressing PD-1 as well as an abundance of PD-L1 expression on tumor cells and leukocytes 2. Unlike the I-E TIME, tumors with a I-I TIME are considered to be immunologically “hot” tumors. Lastly, the TLS TIME is a subclass of the I-I TIME and contains tertiary lymphoid structures (TLSs). The cellular composition of TLSs includes a substantial diversity of lymphocytes, such as B cells, Tregs and DCs, which are similar to the diversity of lymphocytes in lymph nodes 27. The TIME has also been classified based on the presence or absence of tumor infiltrating lymphocytes (TILs) and PD-L1 expression: type I (PD-L1 positive with the presence of TILs, driving adaptive immune resistance), type II (PD-L1 negative with absence of TILs, indicating immune ignorance), type III (PD-L1 positive with absence of TILs, indicating intrinsic induction), and type IV (PD-L1 negative with the presence of TILs, indicating the role of other suppressors in promoting immune tolerance) 28. This proposed classification may be important for designing optimal immunotherapeutic strategies 28, 29. In addition, Thorsson et al. 30 identified six TIME subtypes in 33 diverse cancer types based on the differences in macrophage or lymphocyte signatures, Th1:Th2 cell ratio, extent of intratumoral heterogeneity, aneuploidy, extent of neoantigen load, overall cell proliferation, expression of immunomodulatory genes, and prognosis: wound healing, IFN-γ-dominant, inflammatory, lymphocyte-depleted, immunologically quiet, and TGF-β-dominant. These different TIME classification methods further stratify patients to better understand overall survival and response to immunotherapy.

## Analytical techniques used to characterize the TIME

The complex interactions between tumor cells and the TIME serve as the biological basis for determining the success of immunotherapy. Comprehensive analysis of the landscape of the TIME can be conducted to identify biomarkers that reliably predict the response to therapy and reveal new insights for the development of more effective cancer treatments. Technological advances in studying the TIME have emerged as a crucial area of research at the intersection of cancer biology and immunology. These technological developments have allowed researchers to gain a deeper understanding of the dynamic interactions between tumor cells and the immune system. In this section of the review, we discuss the technological advancements in TIME, including cytometry-based techniques, multiplexing imaging techniques, RNA sequencing, and AI. A summary of the above-mentioned techniques is provided in Table 1.

### Cytometry-based Techniques

#### Flow cytometry (FCM)

**Figure 2:**
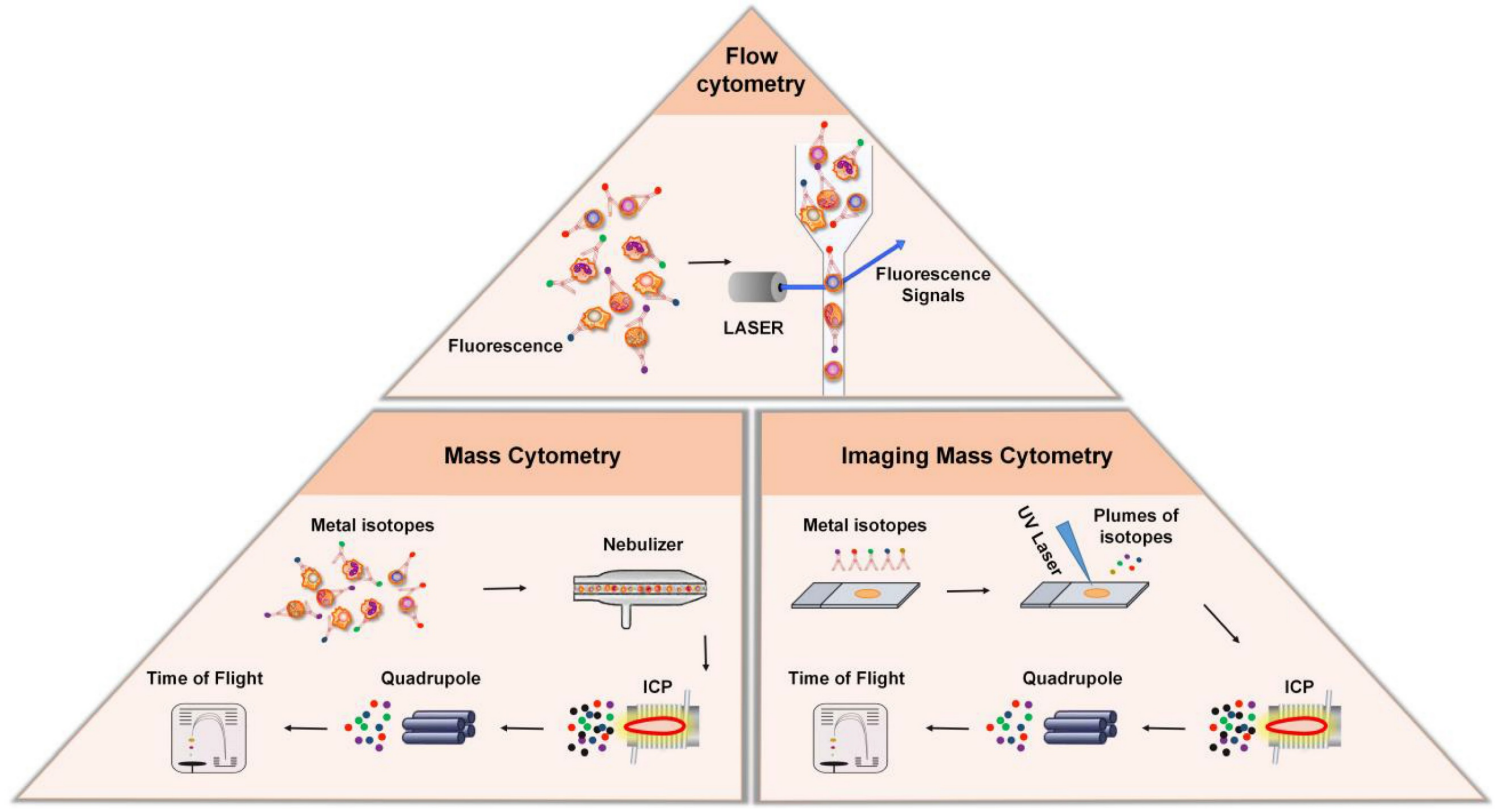
** Cytometry-based techniques. Top:** In flow cytometry, cells are stained with fluorescent dye-labeled antibodies, which bind to certain protein markers. Under the excitation of laser beam, the protein marker emits light, and the antigen density on each cell is determined by measuring the fluorescence signal. **Bottom Left:** Mass cytometry, also known as CyTOF, is a technique that combines flow cytometry and mass spectrometry. During analysis, the cells stained with the metal isotope-conjugated antibodies are passed through a nebulizer, which converts the sample into single-cell droplets. The droplets are subsequently atomized and ionized inside an ICP torch, resulting in the generation of a cloud of metal ions for analysis by time-of-flight mass spectrometry. **Bottom Right:** Imaging mass cytometry is established based on CyTOF and IHC. In this technique, metal-conjugated antibodies label targets in tissue sections, and laser ablation releases metal ions from labeled biomarkers. These ions are detected by the mass spectrometer, enabling spatial mapping and quantification of biomarkers in the tissue.

Flow cytometry (FCM) is an analytical technology that can achieve high-speed, one-by-one quantitative analysis and sorting of cells by detecting the signals emitted from fluorescent molecules with which single cells or other biological particles in suspension are labelled. The advent of FCM has completely revolutionized immunology by enabling the analysis of single cells with multiple parameters. In FCM, cells are stained with fluorescent dye-labeled antibodies, which bind to certain protein markers for which they exhibit high specificity. FCM utilizes highly focused lasers as light sources to generate both scattered and fluorescent light signals (Figure 2). Under excitation with a laser, the protein markers emit light, these signals are then detected by sensitive photodiodes or photomultiplier tubes, and the antigen density on each cell is determined by measuring the corresponding fluorescence signal that is emitted 31. Based on this principle, FCM is widely used in cell classification and immune cell subgroup analysis and plays an important role in the evaluation of human cellular immune function and the diagnosis and treatment of various hematological diseases and tumors. Another major application of FCM is to sort cells according to subtype or epitope expression for further biological studies by using an electrical charge to separate cells based on fluorochrome emission 31.

#### Mass Cytometry

Although FCM is widely used in single-cell labeling and sorting technique, the overlap of the excitation and emission spectra between fluorescent dye-labeled antibody signals limits the number of detected markers 32. New methods, like mass cytometry—also called cytometry by time-of-flight mass spectrometry (CyTOF)—was developed to overcome this limitation 8. In contrast to conventional FCM, CyTOF relies on rare element isotopes, specifically stable metal isotopes, that are conjugated to monoclonal antibodies 8, 33, 34. Because these rare element isotopes are usually absent in cells, their purity and accurate detection by mass spectrometry significantly expand the number of phenotypic markers and improve the resolution of identifiable immune subgroups. During analysis, the cells stained with the metal isotope-conjugated antibodies are passed through a nebulizer, which converts the sample into single-cell droplets (Figure 2). The droplets are subsequently atomized and ionized inside an inductively coupled plasma (ICP) torch, resulting in the generation of a cloud of metal ions. These ions are then analyzed using time-of-flight mass spectrometry, which measures the time it takes for the ions to travel to the detector based on their mass-to-charge ratio (*m/z*). This allows for the detection and quantification of each metal-tagged antibody, the latter of which represents the expression level of the corresponding marker on each individual cell.

CyTOF offers several advantages over traditional FCM, especially high-dimensional analysis and reduced spectral overlap 8, 34. As stated previously, CyTOF utilizes metal isotopes as detection markers instead of traditional fluorophores; this significantly increases the number of measurement parameters and minimizes spectral overlap, allowing for improved resolution and accuracy in data analysis. At present, CyTOF can measure more than 40 parameters at the same time, and the increased resolution has improved the phenotypic diversity of TIME. Zhang et al. 35 identified 18 unique cell populations and defined distinct subsets of CD8^+^ T cells in rheumatoid arthritis joint synovial tissues by integrating mass cytometry and transcriptomics. Hata et al. 36 performed mass cytometry to identify characteristic immune cell subsets in bronchoalveolar lavage fluid from interstitial lung diseases by using two panels including 64 markers. Furthermore, there is minimal or no spectral overlap in CyTOF, thereby reducing or eliminating the need for compensation calculations. CyTOF also has the advantage of enhanced detection sensitivity. Metal tags used in CyTOF have low cellular background signals, resulting in lower noise levels and improved detection of rare cell populations. Levine et al. 37 elucidated a unique metabolic state in rare early-activated T cells by using CyTOF, displaying maximal expression of glycolytic and oxidative metabolic proteins. Their findings demonstrate the effectiveness of mass cytometry in identifying and characterizing rare immune cell populations. However, the use of metal tags also poses several challenges 8, 38. The throughput of mass cytometers is typically limited to around 1,000 cells per second. In addition, the cell injection and cleaning procedures can be time-consuming, adding to the overall run time for each sample. Furthermore, CyTOF does not have the capability to measure forward scatter and side scatter, which are used in traditional FCM to measure cell size and granularity. Cells also cannot be recovered and sorted for subsequent functional analysis, as they are in traditional FCM, and the use of metal tags and specialized instrumentation in CyTOF can result in higher costs compared to traditional FCM.

#### Imaging Mass Cytometry (IMC)

Conventional FCM and CyTOF have changed our ability to understand the phenotype of immune cells, but these technologies require the separation of solid tissue, so they cannot provide the spatial information in the tissue microenvironment. IMC combines an imaging system with a mass spectrometer to construct spatial mapping of isotopes, enabling researchers to obtain insightful information regarding the spatial distribution and abundance of specific biomarkers within the tissues 39-42. The metal-conjugated antibodies bind to their respective targets within the tissue sections, allowing for the specific recognition and labeling of different cell populations and biomarkers (Figure 2). A laser is then used to ablate the tissue section, causing the release of metal ions from the labeled biomarkers. The released metal ions are then captured and transferred to the mass spectrometer, which acquires the mass spectra of the sample, from which the metal-tagged antibodies are identified and quantified. The imaging is performed sequentially, scanning the tissue section pixel-by-pixel, generating a series of mass spectrometry images that represent the distribution of different metal-conjugated antibodies within the tissues. These images can then be analyzed and visualized to study the cellular composition and spatial organization of the tissue.

Overall, IMC can generate high-dimensional tissue spatial images within complex tissue samples 39-41. One of the key advantages of IMC is its compatibility with standard histopathology techniques, making it applicable to various sample types laid on glass slides 43. Furthermore, IMC offers high subcellular resolution, enabling precise localization of proteins within the nuclear, cytoplasmic, and membranous cell compartments 43. Moldoveanu et al. 44 utilized IMC to simultaneously analyze the expression of 35 protein markers at subcellular resolution, enabling the characterization of in-depth spatial quantification of cell-cell interactions within the melanoma microenvironment. Based on IMC, they identified specific cell subpopulations that were associated with positive response to immune checkpoint inhibitors (ICIs). However, the speed of data acquisition with IMC is relatively slow, taking approximately 100 minutes per 1 mm^2^ of tissue, which poses challenges when acquiring data for large or whole slide areas efficiently and cost-effectively 43. The cost of IMC analysis is directly related to the number and volume of metal-tagged antibodies used in co-incubation as well as the surface area of tissue sections to study. These factors contribute to the overall expensive nature of IMC.

### Multiplexing Imaging Techniques

H&E and IHC remain the gold standard for pathological diagnosis and identification of tumor biomarkers in tissues. However, due to the limitation of labelling only one single marker or limited number of markers at once within one tissue section, conventional IHC cannot assess the complex TIME to understand in-depth the relative spatial distribution of immune cells. Although this problem can be solved by consecutive tissue section staining, high-dimensional co-expression analysis cannot be carried out, which will lead to the loss of valuable information 45. To overcome this, mIHC/IF is an emerging technology that provides high-throughput multiplex staining and standardized quantitative analysis for the study of spatial TIME in limited tissue samples 46, 47.

#### mIHC

**Figure 3:**
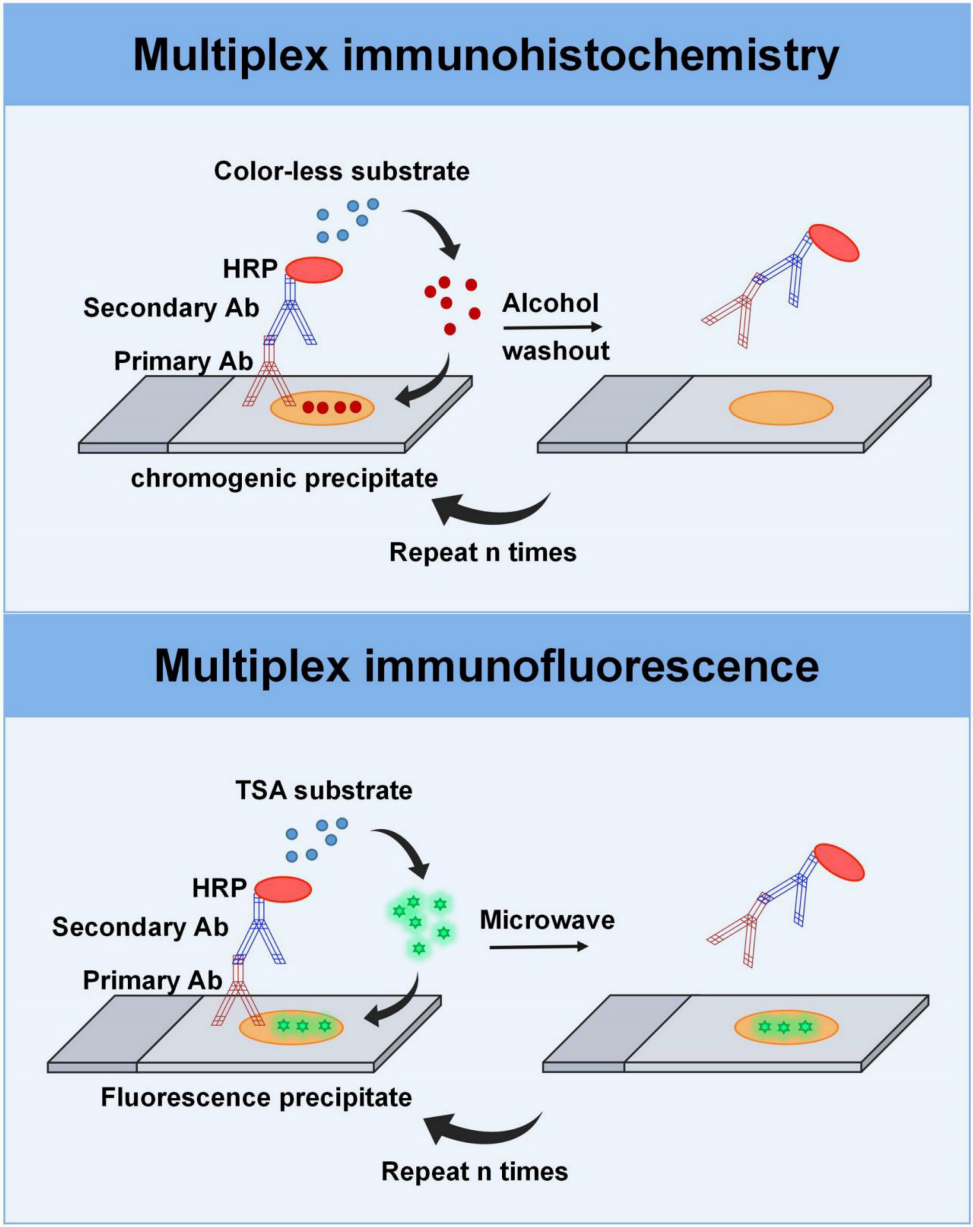
** Multiplexing imaging techniques. Top:** The multiplex immunohistochemistry technique involves a sequential process in which FFPE tissue sections are treated with primary antibodies, followed by secondary antibodies, streptavidin-HRP, and the peroxidase substrate AEC. The stained sections are then counterstained and scanned. After image acquisition, the AEC substrate is removed with the de-staining buffer, allowing for subsequent rounds of staining with different antibodies. **Bottom:** Multiplex immunofluorescence based on TSA provides signal amplification through the combination of HRP detection system and the activation of tyramide fluorophores. Before the next round of staining, the tissue section is heat-treated to remove the non-covalently bound primary and secondary antibodies, while the TSA-conjugated fluorophore is still deposited on the section. After that, the tissue section is incubated in another primary antibody specific for another target and visualized by a different tyramine-linked fluorophore.

mIHC offers the unique capability to label more markers on a single tissue section, expanding the clinical applications to pathologic diagnosis, biomarker research, and immune cell subgroup analysis across various tumor types 48-51. Multiplexed immunohistochemical consecutive staining on a single slide (MICSSS) and sequential immunoperoxidase labelling and erasing (SIMPLE) are main types of mIHC assays that enable sequential staining with multiple dyes using iterative cycles of antigen labeling, image scanning, and decolorization of chromogenic substrate on a single slide 52, 53. In this process, the peroxidase substrate 3-amino-9-ethylcarbazole (AEC) is removed with a de-staining buffer after image acquisition, after which the section is re-stained with a new antibody for other markers of interest, ensuring that MICSS and SIMPLE can carry out multiple rounds of staining (Figure 3). Finally, the images collected after the stainings are overlaid and sometimes even converted into pseudo-color images. Based on this principle, these two technical methods have no restrictions on the types of antibodies. Operationally, MICSSS and SIMPLE are nearly the same as routine IHC workflows, requiring no specific reagents or devices from any particular company, which is highly advantageous for routine clinical pathology laboratories. A potential drawback of the repetitive destaining/restaining method is the possibility of altering tissue integrity and antigen expression. However, one advantage of MICSSS is that it does not compromise antigenicity or create steric hindrance 54. It is important to note, though, that both methods allow for the marking of only one marker in each round of staining, resulting in a limited total number of markers, typically ranging from 5 to 10 53.

#### mIF

mIF is a commonly used technique that allows for the detection of multiple protein targets by staining samples with different fluorophore-labeled antibodies. Compared to mIHC, mIF expands the number of staining markers from approximately 6 to 60 54. Like mIHC, mIF allows sequential staining rounds, but more than one marker can be stained simultaneously during each staining, making mIF more effective and faster than mIHC. The visualization of the primary antibody in mIF can be realized by direct or indirect fluorophore labeling. These antibodies can be labeled with reactive fluorophores, quantum dots, DNA barcodes, etc. 55-58.

Depending on the underlying principles, mIF can be classified into four categories: stain removal technologies, fluorophore inactivation technologies, multiplexed signal amplification, and DNA barcoding technologies 59. Stain removal technologies, like multi-epitope-ligand cartography (MELC), uses photobleaching to remove staining. A recently developed imaging system, known as MACSima Imaging Cyclic Staining (MICS) technology, utilizes a mild signal erasure mechanism, which is achieved by photobleaching the dye or disrupting the labeling conjugate with the release reagent 60. Fluorophore inactivation technologies, such as t-Cycif and MxIF techniques, uses chemical inactivation to eliminate the fluorophore. For example, the MILLAN approach employs SDS/ß-mercaptoethanol washing procedures to completely eliminate antibodies, denaturing and inactivating them, thereby circumventing potential issues stemming from antibody crowding or steric hindrance 61. In recent years, technologies using microfluidics, like the MACSima instrument for MICS, the COMET system from Lunaphore, and the CODEX system from Akoya, have gradually enhanced the automation of cyclic procedures, further expediting the acquisition of a small number of slides 62, 63. CODEX is a multiplexed imaging technique that utilizes DNA-barcoded antibodies for target detection 64, 65. The principle involves staining the tissue sample with DNA-barcoded antibodies, followed by the addition of fluorescence-labeled oligonucleotides that are complementary to those conjugated oligonucleotides. Subsequently, cyclic imaging is performed to capture images for each barcode. CODEX enables the detection of numerous targets in a single sample (no limitation by the number of different antibody species), facilitating the study of complex biological processes and heterogeneity. Additionally, it offers high-resolution imaging, allowing for precise localization and analysis of multiple biomarkers within the tissues. However, it is time-consuming and even requires specialized equipment and expertise. Additionally, it has higher costs compared to traditional methods.

One of the most widely used methods in mIF is indirect multiplexed signal amplification based on tyramide signal amplification (TSA). This method involves the utilization of individual TSA-conjugated fluorophores to detect various targets 47, 55, 66. It provides signal amplification using a combination of polymer-horseradish peroxidase (HRP) and tyramide fluorophores (Figure 3). In the process, after the primary antibody is incubated, the HRP-conjugated secondary antibody is introduced into the sample. Then, the TSA-conjugated fluorophore, which is used as the substrate for HRP, is introduced into the system to generate antigen-related fluorescent signal upon binding to HRP. Before the next round of staining, the tissue section is heat-treated to remove the non-covalently bound primary and secondary antibodies, while the TSA-conjugated fluorophore was still deposited on the section. Following, the tissue section is incubated in another primary antibody specific for another target and visualized by a different tyramine-linked fluorophore. After multiple staining rounds, the signals emitted from the various fluorophores that are bound to the protein targets in the tissue can be extracted according to the wavelength band of a series of scanning images, separated, and quantified by multi-spectral imaging analysis. In addition, the co-expression of various proteins can be characterized in the same sample, enabling protein localization and subcellular analysis. The advantage of this technology is that it allows the use of antibodies from the same species and can simultaneously detect six to eight individual targets, allowing for the generation of an immune cell profile and visualization of complex biological processes 67. For instance, mIF panels can be designed based on various parameters including the cell lineage (such as myeloid or lymphoid), immune state (such as stimulated, pro-inflammatory, and regulatory), and expression of immune checkpoint markers (such as PD-1 and PD-L1) 67.

### RNA sequencing techniques

**Figure 4:**
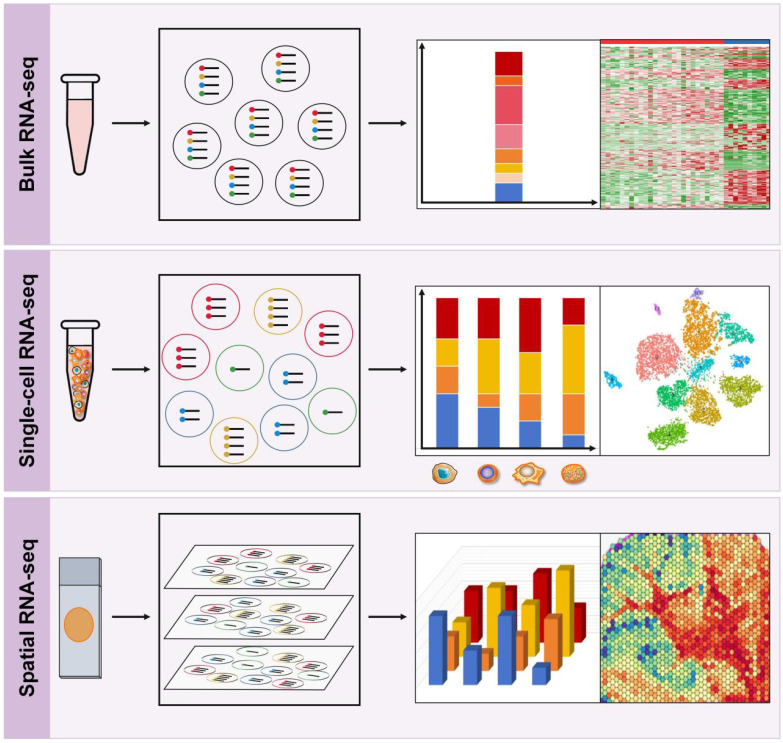
** RNA sequencing techniques. Top:** Bulk RNA-Seq provides an average measure of gene expression across the entire population of cells.** Middle:** Single-cell RNA-Seq enables the analysis of gene expression at the single-cell level, which helps to study cellular diversity and identify unique cell types, providing insights into the complexity and heterogeneity of the TIME. **Bottom:** Spatial RNA-seq is a high-throughput sequencing technique that allows for the profiling of gene expression with spatial resolution in a three-dimensional context within tissue samples.

The genomes within tumors and their microenvironment are promising biomarkers for prognosis prediction. RNA sequencing (RNA-seq) has become a highly useful tool to understand the interactions between cancer cells, immune subgroups, and non-immune interstitial elements, thereby providing a more complete genetic map than DNA sequencing. In recent years, RNA-seq has evolved from classical bulk RNA-seq to scRNA-seq and the emerging spRNA-seq 68. Bulk RNA-Seq provides an average measure of gene expression across the entire population of cells, while scRNA-Seq enables the analysis of gene expression at the single-cell level, and spRNA-seq allows for the study of gene expression in a three-dimensional context, representing the next generation of RNA sequencing (Figure 4).

#### Bulk RNA sequencing

Bulk RNA-seq is the most widely used technique for measuring gene expression at the bulk sample level and has revolutionized the field of immunology research (Figure 4). It enables the comprehensive profiling of the expression of thousands of genes simultaneously, thereby providing insights into the immune transcriptional landscape in response to various stimuli. Bulk RNA-seq analyzes two types of RNA libraries: mRNA-only libraries and whole transcriptome libraries 68, 69. The first type is the most commonly used bulk RNA-seq and is used to profile different gene expression levels to understand the molecular mechanisms or guide diagnosis and treatment. This type of sequencing focuses on mRNA only and requires single-read sequencing (1 × 50 or 1 × 75) at 20-30 million reads/sample, which is simple and cost-effective 69. The second type of bulk RNA-seq analyzes all RNA species in a sample, including mRNA, non-coding RNA (ncRNA) species such as transfer RNA (tRNA), riboswitches, ribozymes, long non-coding RNA (lncRNA), and microRNA (miRNA); however, it does not analyze ribosomal RNA (rRNA) 69. This method requires paired-end sequencing (2 × 100 or 2 × 150) at 40-50 million reads/sample from each direction in order to obtain more comprehensive gene expression information. It allows researchers to study RNA processing and splicing, as well as identify novel transcripts and isoforms.

Bulk RNA-seq provides a comprehensive view of the transcriptional landscape, enabling its broad application in tumor diagnosis, discovery of prognostic biomarkers, identification of novel gene fusions, and guidance for therapeutic interventions 68, 69. Using bulk RNA-seq, numerous novel gene fusions have been identified and utilized as diagnostic or prognostic markers and therapeutic targets in tumors, such as the *NUP98-PHF23* fusion gene in acute myeloid leukemia (AML), as well as recurrent or pathogenic fusion genes like *ESR1-CCDC170*, *SEC16A-NOTCH1*, *SEC22B-NOTCH2*, and *ESR1-YAP1* in breast cancer 69-71. The discovery of some novel fusion genes has even subsequently led to the development of clinical trials for targeted fusion gene drugs, providing new therapeutic opportunities for patients with these fusion genes 69, 72. However, this technique also has some limitations and drawbacks. Bulk RNA-seq measures the gene expression of a heterogeneous population of cells, making it difficult to identify the expression of individual cell types present in the sample.

#### Single-cell RNA sequencing

scRNA-seq refers to a collection of techniques used to analyze the transcriptome of individual cells at a genome-wide level (Figure 4). This technology has revolutionized our ability to investigate cellular diversity and identify unique cell types, providing unprecedented insights into the complexity and heterogeneity of several tumors, such as gastric cancer, bladder cancer, and breast cancer, to name a few, while also enabling the discovery of potential therapeutic targets and biomarkers for patient stratification 38, 73-75. scRNA-seq has also generated vast amounts of data that can be used to trace lineage development in subsets of immune cells, including exhausted T cells, TAMs, DCs, and other lineages. Recent studies have integrated bulk transcriptome and scRNA-seq to provide a more comprehensive understanding of gene expression, cell types, and cellular processes, enabling a systems-level view of immune response dynamics and immune landscape characteristics 76-78.

To date, several scRNA-seq techniques have been employed for sequencing the transcriptome at the single-cell level. One prominent distinction among these scRNA-seq techniques is that some of them can generate full-length or nearly full-length transcript sequencing data, while others focus on capturing and sequencing either the 5'-end or 3'-end of the transcripts 79. Full-length scRNA-seq allows for the comprehensive sequencing of entire RNA transcripts in individual cells. It provides a detailed and unbiased view of gene expression, identifying transcript isoforms, alternative splicing events, allelic expression patterns, and RNA editing events 79. Furthermore, in comparison to 3'-end sequencing techniques, full-length scRNA-seq approaches have the potential advantages in detecting lowly expressed genes or transcripts 80.

There are several techniques available for performing full-length scRNA-seq. Examples of these techniques include the Quartz-seq, “single-cell universal poly(A)-independent RNA sequencing” (SUPeR-seq), “multiple annealing and dC-tailing-based quantitative single-cell RNA-seq” (MATQ-seq), and “switching mechanism at 5'-end of the RNA transcript sequencing” (Smart-Seq) 79. Among them, Smart-Seq is widely recognized as one of the most reliable methods for full-length RNA-seq 81. It leverages the unique characteristic of the Moloney murine leukemia virus reverse transcriptase (MMLV-RT), which exhibits a preference for selecting full-length cDNAs as substrates for its terminal transferase activity. This property enables the coupling of reverse transcription (RT) and template switching (TS) within a single reaction 81. Additionally, the Smart-seq protocol incorporates the design of special primers, ensuring the use of identical primers for cDNA synthesis, which aids in maintaining consistent PCR amplification efficiency 82. Collectively, these attributes contribute to the successful synthesis of full-length cDNA through the Smart-seq method. Smart-seq2 is an enhanced iteration of the original Smart-seq method and is developed to detect a higher number of genes 82. It addresses the issue of 3' bias commonly encountered in many sequencing methods 80. Recently, Smart-seq has evolved into its third generation, referred to as Smart-seq3 83. This upgraded version integrates full-length transcriptome coverage with a 5' unique molecular identifier (UMI) RNA counting strategy 83. This innovative approach enables the in-silico reconstruction of thousands of RNA molecules per cell, leading to enhanced sensitivity compared to Smart-seq2 83. Additionally, Smart-seq3 is capable of detecting longer transcripts at a lower cost.

Notably, full-length sequencing techniques typically have lower throughput, limiting the number of cells that can be sequenced in a single experiment. However, 3'-end-counting scRNA-seq techniques, such as the “cell expression by linear amplification and sequencing” (CEL-seq), “single-cell RNA barcoding and sequencing” (SCRB-seq), “massively parallel RNA single-cell sequencing framework” (MARS-seq), and Drop-seq, capture only the 3'-end of mRNA 79. This process involves the addition of a UMI to the 3' end of each cDNA molecule during reverse transcription, followed by library preparation and sequencing. These sequencing methods focus on the most informative region of the transcript—the 3' end—which contains the majority of the coding sequence and is highly correlated with gene expression levels, enabling the gene expression profiling of a large number of individual cells while providing efficient and cost-effective sequencing data 79, 82.

Recently, microfluidics has played a significant role in advancing scRNA-seq by substantially reducing costs and significantly increasing the throughput of single-cell analysis 84. Microfluidics is a technology that involves the manipulation and control of small volumes of fluids in microscale channels or chambers. Currently, droplet-based RNA-seq technologies, such as Drop-seq, inDrop, and the 10× Genomics Chromium system, as well as high-density microwell-based methods like Seq-well and Microwell-seq, have emerged as the predominant approaches for cell isolation 84, 85.

The 10× Genomics Chromium system is a commercially available droplet-based system for scRNA-seq that utilizes microfluidic chip technology to create a single-cell reaction. This involves the formation of nanoliter-scale aqueous compartments, known as droplets, through the precise combination of aqueous and oil flows in the microfluidic device. Thousands of cells are partitioned into nanoliter-scale Gel Bead-In-Emulsions (GEMs), where the cDNA generated from each cell shares a common cell barcode 86-89. The barcode information can then be used to reconstruct the original cellular identities and analyze gene expression or genomic data at a single-cell resolution. This system offers a streamlined workflow that combines single-cell isolation, amplification, and library preparation, enabling the analysis of a high number of individual cells simultaneously. This allows for increased throughput and improved accuracy in single-cell studies. The system demonstrates a high capture efficiency of up to 65% for single cells, ensuring reliable and robust data collection 90. Additionally, the probability of capturing multiple cells within a single droplet is extremely low, further enhancing the system's ability to achieve true single-cell capture 90. One of the advantages of the 10× Genomics Chromium system is its ability to capture multiple modalities of data from each single cell, including gene expression, surface protein markers, and chromatin accessibility 91. This multi-omics approach allows for a more comprehensive understanding of cellular function and heterogeneity.

Additionally, the 10× Genomics Chromium system has enabled researchers to analyze complex tissues and cellular interactions, such as in the study of the immune system and cancer in TIME. It has been used to investigate the dynamics of immune cell populations, identify novel cell subtypes, and understand how tumors evade the immune system 92. Recent studies have demonstrated the potential of the 10× Genomics Chromium system to characterize the immune landscape of various types of cancer, such as melanoma, lung cancer, and hepatocellular carcinoma (HCC) 91, 93, 94. For example, in melanoma, it has been used to identify a subset of T cells with unique features that are associated with a favorable response to immune checkpoint inhibitors 91. However, it is important to note that the 10× Genomics Chromium system has its limitations. One of the limitations is its high sample requirements. A starting cell quantity of 10^5^-10^6^ cells is required for each sample, and the percentage of viable cells needs to exceed 80% 90, 95. These requirements can present challenges when working with limited or low-quality samples. In addition, scRNA-seq is subject to batch effects, where variation in sample processing and sequencing can result in biased data 96.

The BD Rhapsody platform is another commercial system used for scRNA-seq; however, unlike the 10× Genomics system, it utilizes magnetic beads rather than GEMs in a microfluidic device for cell isolation and barcoding 97-99. Another notable characteristic of the BD Rhapsody system is that it is a microwell-based technology, whereas the 10× Genomics utilizes a droplet-based approach 99. The size and depth of the microwells are carefully optimized in order to minimize the occurrence of double occupancy of beads 99. Moreover, the BD Rhapsody system is equipped with an optically clear window, enabling researchers to visually inspect the contents of the cartridge and each individual microwell. This feature enables quality control and allows researchers to ensure the accuracy of each microwell during the experiment. In addition to the aforementioned advantages, BD Rhapsody beads can be stored for later use, allowing researchers to preserve captured cDNA molecules for several months. The beads also remain intact throughout the workflow, enabling the creation of multiple sequencing libraries by subsampling.

#### Spatial RNA-seq

Despite the numerous advantages of scRNA-seq, it requires lysing individual cells, which can lead to loss of spatial and/or temporal information 38. SpRNA-seq is a high-throughput sequencing technique that allows for the profiling of gene expression with spatial resolution in tissue samples (Figure 4). This technology enables the comprehensive analysis of the TME and characterization of the spatial tumor heterogeneity. In a recent study, the spatiotemporal immune landscape of colorectal cancer liver metastases was investigated at single cell level 100. The authors identified the presence of highly metabolically activated immunosuppressive MRC1^+^ CCL18^+^ M2-like macrophages in the metastatic sites, suggesting that the TIME had undergone significant spatial reprogramming during metastasis 100. Moreover, spatial RNA-seq has greatly advanced the development of more precise and sensitive biomarkers at the molecular, cellular, and microstructural levels, enhancing the optimization of immunotherapy. For example, Zugazagoitia et al. 101. employed a digital spatial profiling (DSP) system to identify numerous pertinent candidate immune predictors in the spatial context and validated that elevated CD56^+^ immune cell counts in the stroma serve as a spatial biomarker that is associated with favorable outcomes in PD-1 checkpoint blockade. Now, 10× spatial transcriptomics and DSP are widely used commercial spRNA-seq techniques.

The 10× spatial transcriptomics technology combines the advantages of microarray analysis and the barcoding system of 10× Genomics to obtain spatially resolved transcriptomic information 68, 102. The workflow begins by imaging the tissue section placed on a Visium slide functionalized with printed oligo capture probes. The Poly-T tails of these capture probes bind the Poly-A tails of RNA molecules. After the tissue section is fixed, stained, and imaged, the tissue sections are permeabilized with a permeabilizing reagent that creates small holes in the cellular membrane, enabling RNA molecules to exit the cells and bind to the adjacent capture probes on the chip. Once captured, the RNA molecules can then undergo reverse transcription on-slide to generate cDNA fragments carrying spatially-defined barcodes. The resulting cDNA is denatured, and the second strand cDNA is collected for off-slide library preparation. The libraries are then sequenced, and bioinformatic analysis is performed to map the reads to specific locations on the tissue. It is a powerful tool for investigating the gene expression patterns and cellular interactions within the TIME. With advancements in technologies to enhance spatial resolution, it is now feasible to identify specific immune cell types and their corresponding gene expression profiles in the TIME. This breakthrough has significant implications in understanding the mechanisms of tumor immune evasion, identifying potential immune targets, and developing new immunotherapeutic strategies. However, there are also limitations to the technology, including the cost and complexity of the workflow, as well as the potential for technical artifacts and biases in data analysis. Nonetheless, the benefits of 10× spatial transcriptomics outweigh the limitations and hold great promise for advancing our understanding of immunology and cancer biology.

DSP, a new generation of spatial multi-target analysis system, integrates tissue image analysis with in situ digital quantitative technology. This method uses oligonucleotide detection technology to obtain the non-destructive, simultaneous high-plex spatial profiling of proteins and RNAs within specific regions of interest (ROIs) on formalin-fixed, paraffin-embedded (FFPE) samples 47, 55, 103. Two types of primary antibodies or RNA-probes are used in this method. Firstly, small oligonucleotide “barcodes” (PC-oligos) are conjugated to primary antibodies or RNA-probes through a photocleavable UV light-sensitive linker for target interrogation. Secondly, fluorophores are conjugated to primary antibodies to enable the identification of different cellular compartments (up to four compartments) or cell markers. Fluorescence microscopy images of the tissue are acquired to understand the morphology of the tissue. Then, an ROI in the sample is identified, and UV light is projected onto the tissue sample to release the PC oligonucleotides from the antibodies or RNA-probes at the defined ROI. After that, the released PC oligonucleotides are automatically collected in tubes and transferred to a microtiter plate to be read by an nCounter analysis system or next generation sequencing (NGS). Then, the data will be mapped back to the previous morphological images to obtain the spatial profiling at the defined ROIs. This high-throughput technology allows for the adoption of non-invasive methods to maintain the integrity of the sample, the detection of highly multiplexed RNA or protein (up to 1800 targets can be detected in one round from a single FFPE section), and the selection of ROIs for high-plex analysis. However, DSP also has some limitations. For example, unlike other multiple staining techniques, DSP cannot generate images. In addition, the selection of ROIs is subjective, which may lead to biased hypothesis-driven sample analysis 104. Furthermore, only four fluorescence channels are used to visualize tissue morphology, which limits the throughput of more morphological details, such as the spatial organization of different types of immune cells in the TIME 104.

### Artificial Intelligence (AI)

**Figure 5:**
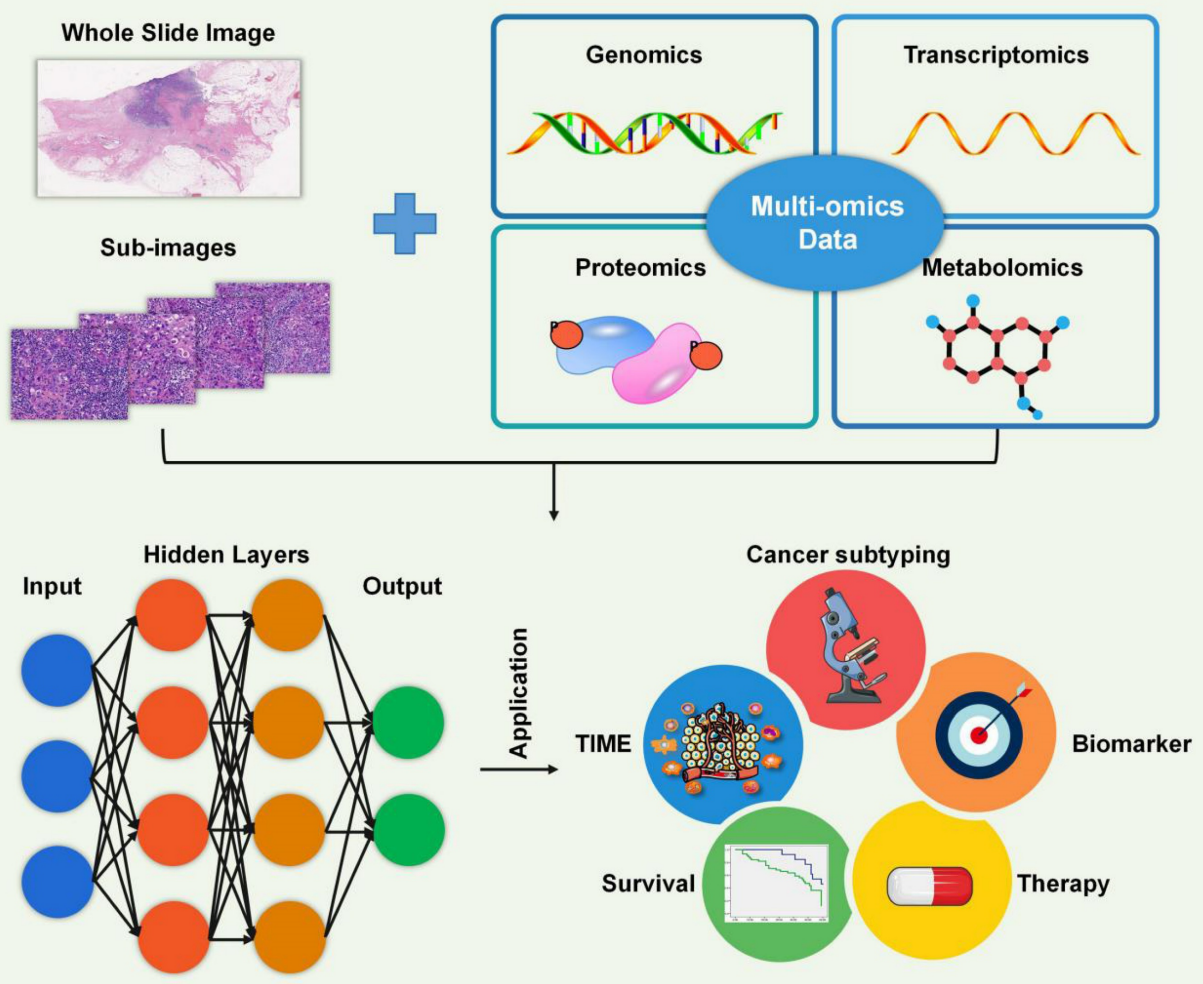
** Artificial intelligence.** Artificial intelligence is a powerful tool for analyzing diverse biological big data, including pathology, genomics, transcriptomics, proteomics, and metabolomics. It is widely used in various areas such as the characterization of the TIME, identification of cancer subtypes, recognition of biomarkers, selection of treatment options, and prediction of patient survival.

In the analysis of tumor samples and associated clinical data, high-throughput technologies generate vast amounts of complex omics data, such as gene expression profiles (transcriptomics), protein expression patterns (proteomics), and genetic variations (genomics). However, analyzing gene expression data from the tumor microenvironment can be complicated, and accurately identifying immune cell infiltrations can be challenging. AI has become a powerful tool in the analysis and interpretation of various types of biological data, including pathology, genomics, transcriptomics, proteomics, and metabolomics (Figure 5). By leveraging machine learning (ML) and deep learning (DL) algorithms, AI has revolutionized the analysis of the TME by enabling the discovery of complex patterns and relationships within large and diverse datasets 105-107.

Machine Learning (ML) is a subset of AI that focuses on use and the development of algorithms and statistical models that enable computers to learn from data and make predictions or decisions without being explicitly programmed 108. ML algorithms can analyze and identify patterns, trends, and relationships within complex datasets, enabling the system to improve its performance over time through experience. ML can also help to classify immune cell types in the microenvironment using unsupervised or supervised machine learning algorithms 109, 110. Unsupervised clustering algorithms, such as t-Distributed Stochastic Neighbor Embedding (t-SNE) or principal component analysis (PCA), can be used to classify cell types based on transcriptome profiles. Supervised machine learning algorithms, such as support vector machines (SVM) or random forests, can identify cell populations and predict functional pathways specific to each. This is crucial in understanding the immune cell types involved in cancer progression and the potential for developing targeted therapies.

Deep Learning (DL) is a specific branch of ML that uses neural networks with multiple layers to model and process complex patterns and representations. Inspired by the structure and function of the human brain, DL algorithms have the ability to automatically learn hierarchical representations from the data and extract high-level features, enabling more advanced and accurate predictions or decision-making 108. DL models, such as convolutional neural networks (CNN), analyze histopathology and immunohistochemistry images to quantify immune cell types, their spatial distribution, and interactions within the tumor microenvironment 111, 112. This aids in characterizing immune cell infiltration patterns and predicting treatment outcomes. Recurrent Neural Networks (RNN) are capable of analyzing sequential data (such as time-series gene expression data) to model the dynamic changes in immune cell populations over time. DL architectures, such as deep belief networks or autoencoders, are employed to integrate and analyze multi-omics data encompassing RNA-Seq, whole-genome sequencing, whole-exome sequencing, or proteomics. This approach facilitates the identification of novel immune signatures associated with the immune microenvironment 113, 114. Deep generative models, such as generative adversarial networks (GANs), can generate synthetic histopathology images or immunohistochemistry images that resemble real tissue samples. These synthetic images can be used for training models, augmenting datasets, or understanding immune cell spatial distribution within the tumor microenvironment 114, 115.

AI models applied to Big Data have made significant contributions to the understanding and treatment of cancer by enabling analysis of the TIME, cancer subtyping, biomarker identification, therapy selection, and survival prediction (Figure 5). In terms of the TIME, AI algorithms can analyze multi-dimensional immune profiling data, including gene expression, immunohistochemistry, and single-cell sequencing, to characterize the immune response within a tumor. A recently developed ML classifier, known as ImmClassifier, demonstrated superior performance in accurately classifying fine-grained immune cell types from single-cell RNA-Seq data, enabling deeper investigations into the extensive heterogeneity of the immune system 110. AI can identify specific immune cell types and their spatial distributions as well as functional states, providing insights into the interactions between tumor cells and the immune system. Saltz et al. 112 developed a DL-derived “computational stain” based on H&E images from 4,759 TCGA subjects to generate a TILs map for identifying spatial heterogeneity patterns of TILs and correlating the TIL patterns with immune profiles, cancer subtypes, and survival outcome 112. This knowledge will assist in understanding the tumor immune landscape, predicting the response to immunotherapies, and developing strategies to overcome immunosuppressive mechanisms. By analyzing histopathological images, AI algorithms can accurately identify cancerous cells, detect specific tissue abnormalities, and predict patient outcomes. AI models have been developed to aid pathologists in detecting and characterizing various diseases, such as lymphoma, breast cancer, and lung carcinoma 116-118. Biomarker discovery is another area in which AI has excelled. By analyzing omics data, AI algorithms can identify potential biomarkers for cancer diagnosis, prognosis, and treatment response 119. For augmenting therapy selection, AI algorithms can integrate patient clinical data, molecular profiling data, and treatment outcomes to predict the most effective treatment strategies for individual patients. By utilizing ML and predictive modeling, AI can assist clinicians in personalized treatment decision-making, optimizing chemotherapy regimens, and identifying appropriate targeted therapies, including immunotherapies 114, 119. AI also contributes to survival prediction by utilizing various clinical and molecular features to estimate patient outcomes. By training on large cohorts with known survival outcomes, AI algorithms can accurately predict patient survival, providing valuable information for treatment planning and prognosis 114, 119.

## Challenges and future prospects

In this review, the investigation of the immune microenvironment has been greatly facilitated by various technologies, however, it is important to acknowledge that these technologies also encounter certain challenges (Table 1). These challenges include limited parameter measurement in cytometry-based techniques and multiplexing imaging techniques, technical variability and high cost associated with RNA sequencing, and the interpretability of AI models. Overcoming these challenges will pave the way for future advancements in TIME research.

To address the challenges in cytometry-based techniques, future developments should focus on increasing the number of measurable parameters, improving resolution and sensitivity, and automating these techniques. Innovative engineering approaches and spectral unmixing algorithms can further enhance our ability to analyze complex immune cell populations. Multiplexing imaging techniques, such as mIHC and mIF, offer the capability to simultaneously visualize multiple biomarkers within complex tissue samples. Despite their potential, challenges such as limited simultaneously detectable markers, spectral overlap, tissue autofluorescence need to be addressed. Future developments may include improving multiplexing capabilities and integrating spatial transcriptomics. In RNA sequencing, technical variability and high costs remain challenges, technical noise and confounding factors hinder subsequent analyses. Future advancements should aim to improve sensitivity, accuracy, and scalability of RNA sequencing technologies. Integration of spatial transcriptomics with imaging techniques and scRNA-seq will provide a more comprehensive understanding of the TIME. AI has shown great potential in analyzing complex datasets. However, challenges persist in obtaining annotated datasets, interpreting AI models, and addressing ethical considerations. Future developments may involve the integration of AI with cytometry-based techniques, multiplexing imaging techniques, and RNA sequencing data. DL models may be refined and explainable AI approaches may be developed to enhance the interpretability and reliability of AI predictions.

Looking ahead, advancements in technology hold immense promise for immune microenvironment research. Overcoming the current challenges will allow for a more comprehensive understanding of immune responses in health and disease. By further refining and integrating cytometry-based techniques, multiplexing imaging techniques, RNA sequencing, and AI, we can unravel the complexities of the immune microenvironment and pave the way for personalized immunotherapies and improved patient outcomes.

## Figures and Tables

**Table 1:** Summary of techniques in the characterization of the tumor immune microenvironment

Category	Techniques	Advantages	Limitations	Suitable Applications
Cytometry-based Techniques	Flow cytometry (FCM)	l high-speed l relatively high throughput l capable of cell sorting	l spectral overlap l limited number of detected markers l lack of spatial information	l single-cell labeling l cell sorting, counting, viability, and phenotypic analysis l immune cell subgroup analysis
	Mass Cytometry	l high-dimensional analysis (>40 parameters) l reduced spectral overlap and better resolution l minimal compensation requirements l enhanced detection sensitivity	l relatively low throughput l cells cannot be recovered and sorted l higher costs l lack of spatial information	l deep phenotyping of immune cell subsets l identification of rare cell populations
	Imaging Mass Cytometry (IMC)	l high-dimensional tissue spatial imaging l high subcellular resolution l applicable to various sample types laid on glass slides	l time-consuming l very expensive	l subcellular spatial information in the tumor microenvironment l investigation of tissue architecture, cell-cell interactions, and signaling pathways at high resolution
Multiplexing Imaging Techniques	multiplex immunohistochemistry (mIHC)	l simultaneous assessment of multiple biomarkers on the same tissue section l no restrictions on the types of antibodies l simple technique (similar to routine IHC)	l only one marker in each round of staining l limited simultaneously detectable markers (5-10) l time-consuming	l biomarker research l immune cell subgroup analysis
	multiplex immunofluorescence (mIF)	l simultaneous assessment of multiple biomarkers and high-resolution imaging l more staining markers l TSA amplifies fluorescent signal and enhances sensitivity	l spectral overlap and signal bleed-through l background fluorescence l higher costs	l immune cell profiling l protein localization and subcellular analysis
RNA Sequencing Techniques	Bulk RNA sequencing (Bulk RNA-seq)	l a global view of gene expression in a population of cells l high throughput l cost-effective and suitable for large-scale sample analysis	l average gene expression profile, loss of cellular heterogeneity information l lack of spatial information	l transcriptome profiling l tumor diagnosis, prognosis biomarker discovery, novel gene fusion discovery, and guidance for therapeutic treatment
	Single-cell RNA sequencing (scRNA-seq)	l enables the analysis of gene expression at the single-cell level l tumor heterogeneity analysis l identification of unique cell types and rare cell populations	l higher cost l limited sensitivity and higher levels of technical noise l lack of spatial information	l tumor heterogeneity l cell typing and lineage tracing l immune response dynamics and immune landscape characteristics
	spatial RNA sequencing (spRNA-seq)	l provides spatially resolved transcriptome information l 10× spatial transcriptomics: higher cellular resolution l DSP: high-plex gene detection capability	l higher cost and complexity of the workflow l the technique is relatively new and still developing	l spatial tumor heterogeneity l comprehensive analysis of the tumor microenvironment l spatially biomarker discovery l immunotherapy optimization
Artificial Intelligence (AI)		l big data analysis l integration of multi-omics data l efficient and automated analysis	l lack of interpretability l limited availability l ethical considerations	l tumor microenvironment analysis l cancer subtyping l biomarker identification l therapy selection l survival prediction
